# Evaluation of High-Performance Curcumin Nanocrystals for Pulmonary Drug Delivery Both *In Vitro* and *In Vivo*

**DOI:** 10.1186/s11671-015-1085-y

**Published:** 2015-10-01

**Authors:** Liandong Hu, Dongqian Kong, Qiaofeng Hu, Na Gao, Saixi Pang

**Affiliations:** School of Pharmaceutical Sciences, Hebei University, No. 180, WuSi Road, Baoding, 071002 People’s Republic of China; Key Laboratory of Pharmaceutical Quality Control of Hebei Province, Hebei University, Baoding, People’s Republic of China; CSPC Pharmaceutical Group NBP Pharmaceutical Co. Ltd, Shijiazhuang, People’s Republic of China

**Keywords:** Curcumin, Wet milling, Nanocrystals, Pulmonary delivery, Tissue distribution

## Abstract

This paper focused on formulating high-performance curcumin spray-dried powders for inhalation (curcumin-DPIs) to achieve a high lung concentration. Curcumin-DPIs were produced using wet milling combined with the spray drying method. The effects of different milling times on particle size and aerodynamic performance were investigated. The curcumin-DPIs were characterized by scanning electron microscopy (SEM), differential scanning calorimetry (DSC), powder X-ray diffraction (PXRD), Fourier transform infrared spectroscopy (FTIR), and *in vitro* dissolution. Furthermore, the *in vivo* pharmacokinetic behavior and tissue distribution after pulmonary administration were also evaluated. Results showed that the drug dissolution was significantly enhanced by processing into curcumin-DPIs. The aerodynamic results indicated that the DPIs displayed a good aerosol performance. The plasma curcumin concentration was obviously enhanced by inhalation, and most of the curcumin-DPIs were deposited in the lung. This study demonstrated that inhalation was an effective way to carry drug to the lung, and curcumin-DPIs were hopeful for lung cancer treatment in the future.

**Electronic supplementary material:**

The online version of this article (doi:10.1186/s11671-015-1085-y) contains supplementary material, which is available to authorized users.

## Background

Curcumin is a yellow substance isolated from the rhizome of *Curcuma longa* [1], which has extensive pharmacological actions such as antitumor [2], anti-inflammatory [3], and antioxidant effects [4]. Curcumin is pharmacologically safe and is used as a dietary spice and food additive. Some researchers reported that curcumin can effectively inhibit tumor cell proliferation, migration, and invasion [5]. However, the clinical application of curcumin is limited due to its extremely low aqueous solubility, instability in aqueous solution, rapid metabolism, and poor bioavailability [6].

In order to solve this problem, reducing the particle sizes to nanoscale by wet milling is often used. Wet milling is considered to be one of the best approaches to prepare nanocrystals for large-scale production [7]. The mixed coarse suspension of drug and stabilizers are fed into the grinding jar with grinding media. The mechanical attrition is a high-energy process with high-speed shear force and impact force, which can produce suspensions with smaller size. As an efficient and convenient method, wet milling technique can sharply increase the surface area of drug particles and improve the dissolution rate [8], resulting in a better absorption [9].

In recent years, pulmonary drug delivery brings great interest to researchers due to many advantages in both local and systemic treatments over other delivery routes. The lung has relatively large surface areas (43 to 102 m^2^), thin absorption barriers, and low proteolytic activities. The lung has significant blood capillaries to make the drug be absorbed rapidly, and the pulmonary administration could avoid first-pass effect of the liver [10].

 The technology of dry powders for inhalation (DPI) is propellant-free, portable, and easy to operate, and it has better stability for inhalation. DPI can target drug to the lung and result in an effective therapeutic concentration at the pathological site. DPI also provides a sustained effect with a minimal administration dosage, and reduces the frequency of medication and increases the patients’ compliance. DPI requires drug particles with optimum size and good flow property to ensure accurate dose for better inhalation. Spray drying is an ideal technique because it can produce spherical particles with good uniformity and mobility.

In this study, wet milling technique in combination with spray drying was used to prepare curcumin-DPIs and the milling time was optimized. Physicochemical properties of the powders were characterized by differential scanning calorimetry (DSC), powder X-ray diffraction (PXRD), Fourier transform infrared spectroscopy (FTIR), scanning electron microscopy (SEM), and *in vitro* dissolution. The plasma curcumin concentration and *in vivo* tissue distribution were also investigated. Results showed that inhalation was an effective way to carry drug to the lung, and curcumin-DPIs were hopeful for lung cancer treatment in the future.

## Methods

### Materials

Curcumin was obtained from Sinopharm Chemical Reagent Co., Ltd. (Shanghai, China). Tween 80 was purchased from Yibei Chemical Reagent (Tianjin, China). Methanol and ethanol were both provided by Beichen Founder Reagent Plant (Tianjin, China). Acetonitrile was obtained from Kermel Chemical Reagent Co., Ltd. (Tianjin, China). Sodium dodecyl sulphate was purchased from Tianjin Baodi Chemical Holding Co., Ltd. (Tianjin, China). Acetic acid glacial was purchased from Fuchen Chemical Reagent (Tianjin, China). All other chemicals were of analytical grade. Distilled water was used throughout the study.

### Preparation of Curcumin Nanocrystals

The nanocrystals were prepared using a MiniZeta (NETZSCH Machinery and Instruments Co., Ltd., Germany) machine. The grinding media was yttrium-stabilized zirconium oxide bead (0.6 mm in diameter). Before milling, 20 g curcumin was dispersed in 250 mL aqueous solution of 6.25 % Tween 80 (relative to the drug amount) under magnetic stirring, until a relatively uniform coarse suspension was obtained. Then, the coarse suspension was transferred to the milling bowl. The system temperature was maintained at less than 25 °C by passing cooling water through the outer jacket continuously.

### Spray Drying of Curcumin Nanocrystals

The nanocrystals were spray-dried with an YC-015 experimental spray drier (Shanghai, China). The operating condition was set as follows: inlet temperature of 170 °C, outlet temperature of 90 °C, and liquid feed rate of 3 mL/min. A magnetic stirrer was used to keep the suspension homogenized. Dried powders were collected in small bottles and stored in separate desiccators for further study [11].

### Characterizations

#### Particle Size

The particle sizes of samples before and after wet milling were evaluated by the laser diffraction method using a BT-9300S laser particle size analyzer (Dandong, China). Each sample was diluted with purified water to achieve a suitable concentration for measurement. Results were expressed as D_50_, D_90_ and D_97_, which meant 50, 90, and 97 % of the particles were smaller than the rest of the distribution, respectively.

#### Drug Loading

To quantify the curcumin content, a certain amount of curcumin-DPIs was dissolved in ethanol and mixed by ultrasonication for 10 min. The solution was diluted to a suitable concentration with ethanol and filtered through a 0.45-μm filter membrane. The subsequent filtrate was assayed by high-performance liquid chromatography (HPLC) to determine the concentration of curcumin. The HPLC system consisted of two LC-20A pumps and an SPD-20A UV/VIS detector (Shimadzu, Kyoto, Japan). The column used was Thermo C18 (250 × 4.6 mm). Acetonitrile/water (containing 0.05 % acetic acid glacial) (60:40, mL/mL) was used as the mobile phase with a flow rate of 1 mL/min. The wavelength was set at 428 nm.

#### Aerodynamic Particle Size

The aerodynamic particle size was evaluated using a next generation impactor (model 170 NGI, MSP Corporation), which was equipped with a pre-separator [12]. The flow rate was adjusted to 60 L/min. The dry powder inhaler was fitted with a size 3 HPMC capsule. Deposited drugs in the device, throat, and cups were removed with ethanol and collected in volumetric flasks. The cutoff diameters of stages 1, 2, 3, 4, 5, 6, and 7 were 8.06, 4.46, 2.82, 1.66, 0.94, 0.55, and 0.34 μm, respectively. Stage 8 referred to capturing the remaining fractions of particles less than 0.34 μm. Each stage of the impactor was coated with silicone prior to analysis in order to minimize particle bounce. Drug deposition was determined by a validated HPLC method described above. Fine particle fraction (FPF) was used to describe the quality of an inhalation formulation. FPF was calculated as the fraction of a formulation that is in a size range with the potential to penetrate and deposit in the airways. The following formula was used to calculate FPF_loaded_ and FPF_emitted_:

1 $$ {\mathrm{FPF}}_{\mathrm{loaded}}=\frac{\mathrm{powder}\kern0.5em \mathrm{amount}\kern0.5em \mathrm{on}\kern0.5em \mathrm{stages}\kern0.5em 2-8}{\mathrm{total}\kern0.5em \mathrm{amount}\kern0.5em \mathrm{recovered}} $$

2 $$ {\mathrm{FPF}}_{\mathrm{emitted}}=\frac{\mathrm{powder}\kern0.5em \mathrm{amount}\mathrm{on}\kern0.5em \mathrm{stages}\kern0.5em 2-8}{\mathrm{total}\kern0.5em \mathrm{amount}\kern0.5em \mathrm{recovered}-\mathrm{capsule}\kern0.5em \mathrm{and}\kern0.5em \mathrm{device}\kern0.5em \mathrm{retention}} $$

#### DSC, FTIR, and PXRD

DSC was conducted using a DSC822E differential scanning calorimeter (PerkinElmer, USA) to examine the thermal properties of different formulations. The samples were accurately weighed and placed in a crucible with an empty crucible as the reference. The temperature ranged from 32 to 250 °C with a heating rate of 10 °C/min.

FTIR spectra were tested using a FTIR spectrometer (Shimadzu 8400S, Japan). Each sample was mixed with potassium bromide of IR grade and compressed using an IR pellet manufacturing machine.

PXRD was imaged by a Y2000 powder X-ray diffractometer (Dandong, China). The powders were placed in the sample slot and pressed smoothly with frosted glass. Then, samples were put into an instrument with a scan speed at 0.04°/min, and the patterns were recorded over 2*θ*, with the angle ranged from 5° to 50°.

#### In Vitro Dissolution

The dissolution behaviors of bulk curcumin and curcumin-DPIs were investigated by the paddle method using a ZRS-8G dissolution apparatus (Tianjin, China) at a rotation speed of 100 rpm in 900 mL 0.3, 0.5, and 1.0 % sodium dodecyl sulfate (SDS) aqueous solution (g/mL), respectively. The temperature was maintained at 37 ± 0.5 °C. At specific time intervals, 5 mL of samples was withdrawn and immediately replaced with the same amount of fresh media. All the samples were passed through a 0.45-μm filter membrane and then examined with an UV spectrophotometer at 428 nm.

#### Scanning Electron Microscopy

Surface morphologies of bulk curcumin and curcumin-DPIs were investigated by SEM (JSM-7500 F, Japan). Samples were fixed on stubs using a double-sided tape and coated with gold under a high-vacuum atmosphere and then observed at an acceleration voltage of 10 kV.

#### Stability

Curcumin-DPIs were sealed in small bottles and stored at 4 and 25 °C, respectively. The concentration was measured after 60 days. Any decrease in the drug content or occurrence of extra drug impurity in chromatograms would be considered as instability.

#### Pharmacokinetic Study and Tissue Distribution

In this study, 12 rabbits were housed in separate cages and received food and water ad libitum. Twelve hours before experiments, the rabbits were randomly separated into two groups and fasted but with free access to water. Rabbits were anesthetized with chloral hydrate (100 mg/kg, i.v.) and received endotracheal insufflation and oral administration of curcumin-DPIs (25 mg/kg), respectively. Blood samples were taken from the ear vein at predetermined intervals (0, 0.5, 0.75, 1, 2, 3, 4, 6, and 8 h) and collected in heparinized tubes. Total blood was centrifuged at 10,000 rpm for 10 min, and 200 μL of plasma was collected and stored at −20 °C until analysis.

Tissue distribution was studied on Wistar rats (250 ± 20 g). Before the experiments, they were randomly separated into two groups and anesthetized with urethane (1 g/kg, i.p.) and then received pulmonary administration of curcumin-DPIs (25 mg/kg). At determined time points (0.5, 2, 4, and 6 h), the rats were sacrificed, and the tissues including the heart, liver, spleen, lung, kidney, and brain were collected, weighted, and frozen at −20 °C until further analysis.

For plasma samples, 0.6 mL ethanol was added into 0.2 mL plasma, vortexing for 5 min. Then, the mixture was centrifuged to obtain the supernatant. For tissue samples, the tissues were formulated into homogenates using saline solution. 0.5 mL of tissue homogenates were collected and 1 mL methanol was added. The mixture was vortexed for 3 min and centrifuged at 10,000 rpm for 10 min, then the supernatant was collected. Concentrations of curcumin in plasma and tissue samples were measured by a HPLC method mentioned above.

## Results and Discussion

### Effects of Milling Time

**Fig. 1:**
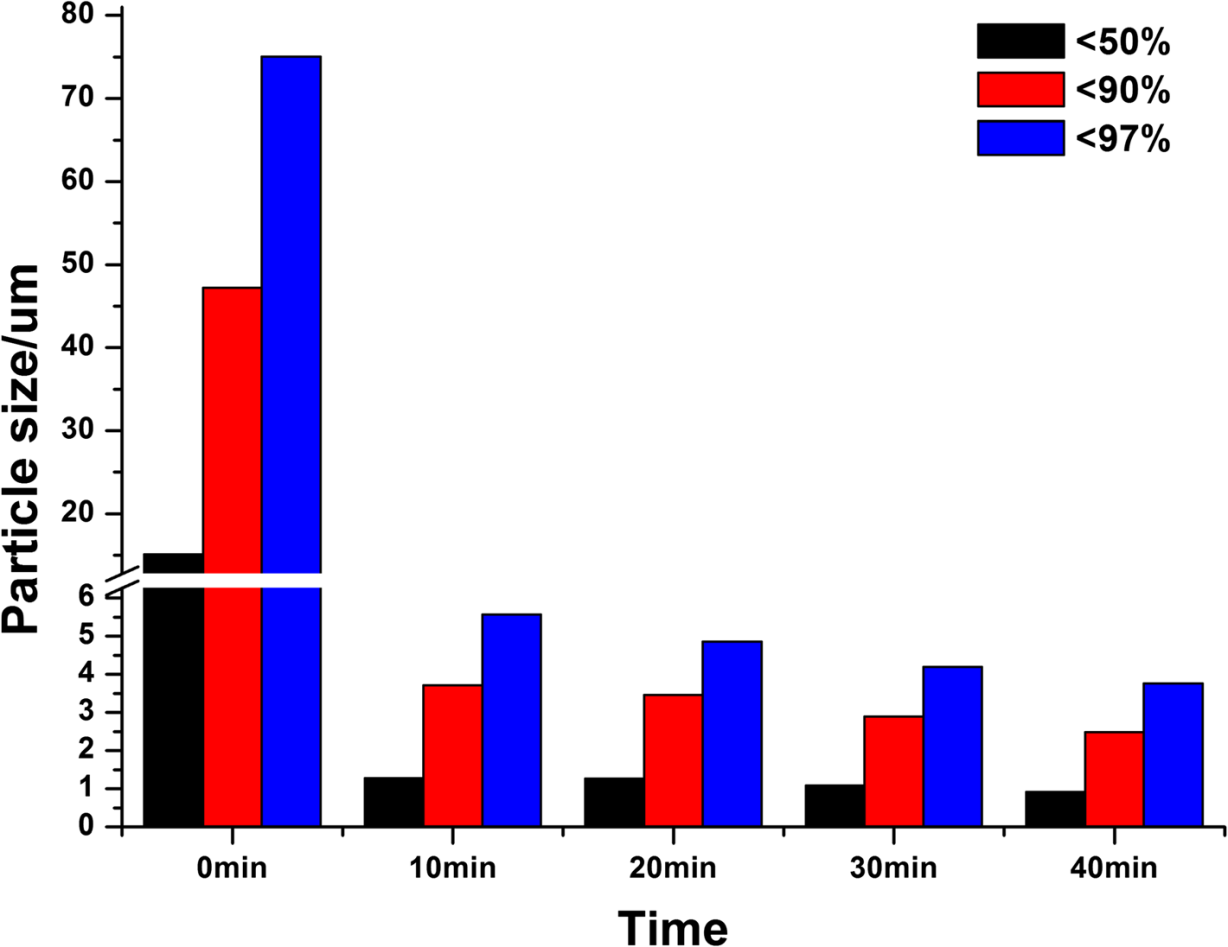
Particle sizes of unmilled bulk curcumin (0 min) and nanocrystals milled with different times

The effects of milling time on particle sizes were shown in Fig. 1. For all cases, the concentration of Tween 80 was kept 6.25 % relative to the drug amount and the agitator speed was 3000 rpm. Before milling, the D_50_, D_90_, and D_97_ of bulk curcumin were 15.08, 47.19, and 75.01 μm, respectively.

It was found that the particle sizes decreased obviously with increasing milling time. After milling for 10 min, the D_50_ of curcumin dramatically decreased to less than 1.5 μm. Nanocrystals with smaller and more uniform particle size were obtained by prolonging milling time. The D_50_ decreased to 1277 nm after milling for 10 min, 1268 nm for 20 min, 1086 nm for 30 min, and 924 nm for 40 min. It could be concluded that extending the milling time would provide more energy to break the crystals into smaller ones and give sufficient spreading time for Tween 80 to attach onto the particle surfaces. In addition, further increasing the milling time to 60 min did not remarkably decrease particle size (Additional file 1).

### Aerodynamic Particle Size

**Fig. 2:**
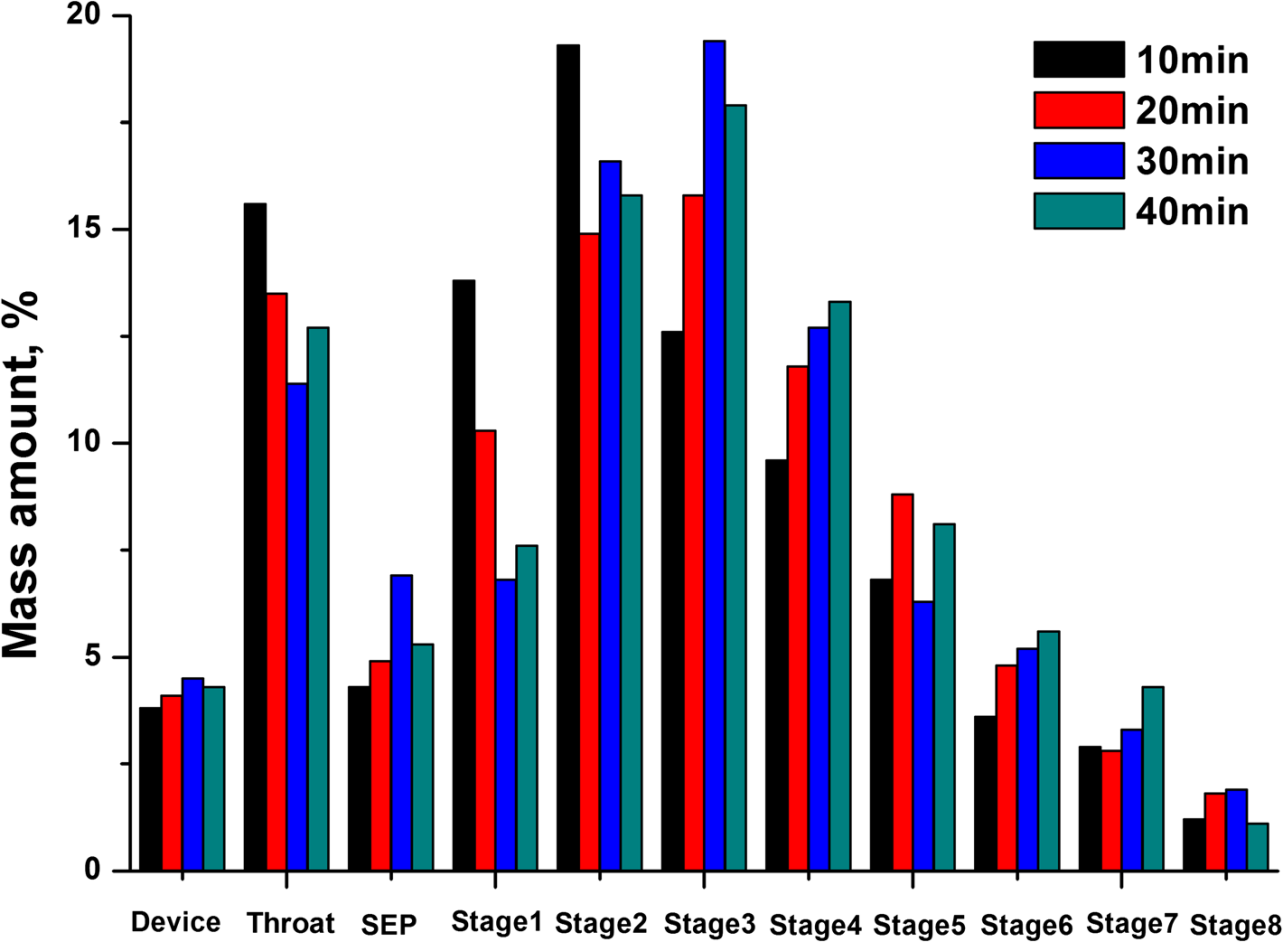
Next generation impactor deposition profiles of curcumin-DPIs milled with different times

The deposition site in the respiratory tract and the inhaled efficiency were critically influenced by particle size distributions. Figure 2 showed the aerosol behavior of the spray-dried powders with different milling times. The FPF_loaded_ and FPF_emitted_ were 59.9 and 62.4 % for 10 min, 64.9 and 67.9 % for 20 min, 68.9 and 72.1 % for 30 min, and 68.8 and 72.3 % for 40 min, respectively (Table 1). The differences in the deposition patterns may be due to the decrease in the particle sizes. Furthermore, it was reported that inhaled particles below 5 μm could be able to penetrate into the lung [13]. So, most of the spray-dried powders could penetrate into the lung, thus increasing the curcumin’s lung deposition. In spite of the higher powder dispersion behavior, the dried powders still had a small amount of capsule and device retention. Powders may adhere in the capsule and the inhalation device during the emission process. Results showed that the powders milling for 30 and 40 min had higher FPF_loaded_ and FPF_emitted_ values and the former had a smaller capsule and device retention. So the milling time was set as 30 min for curcumin-DPI production, which could ensure a good aerosol performance.

**Table 1:** Aerodynamic properties of curcumin-DPIs dispersed at 60 L/min with different milling times

Time (min)	FPF_loaded_ (%)	FPF_emitted_ (%)	Capsule and device retention (%)
10	59.9	62.4	3.8
20	64.9	67.9	4.1
30	68.9	72.1	4.3
40	68.8	72.3	4.5

### Characterizations

#### Thermal Analysis

**Fig. 3:**
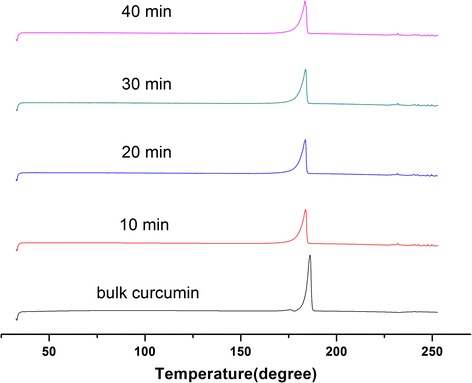
DSC curves of bulk curcumin and curcumin-DPIs milled with different times

In Fig. 3, the bulk curcumin exhibited a sharp endotherm peak with melting point at 186.2 °C, while the melting point of curcumin-DPIs was between 183.2 and 183.7 °C. The melting point of curcumin-DPIs shifted forward with significant reduction in peak intensity, and it may be caused by the interactions between curcumin and the co-grinding surfactant in the experimental process.

#### PXRD Analysis

**Fig. 4:**
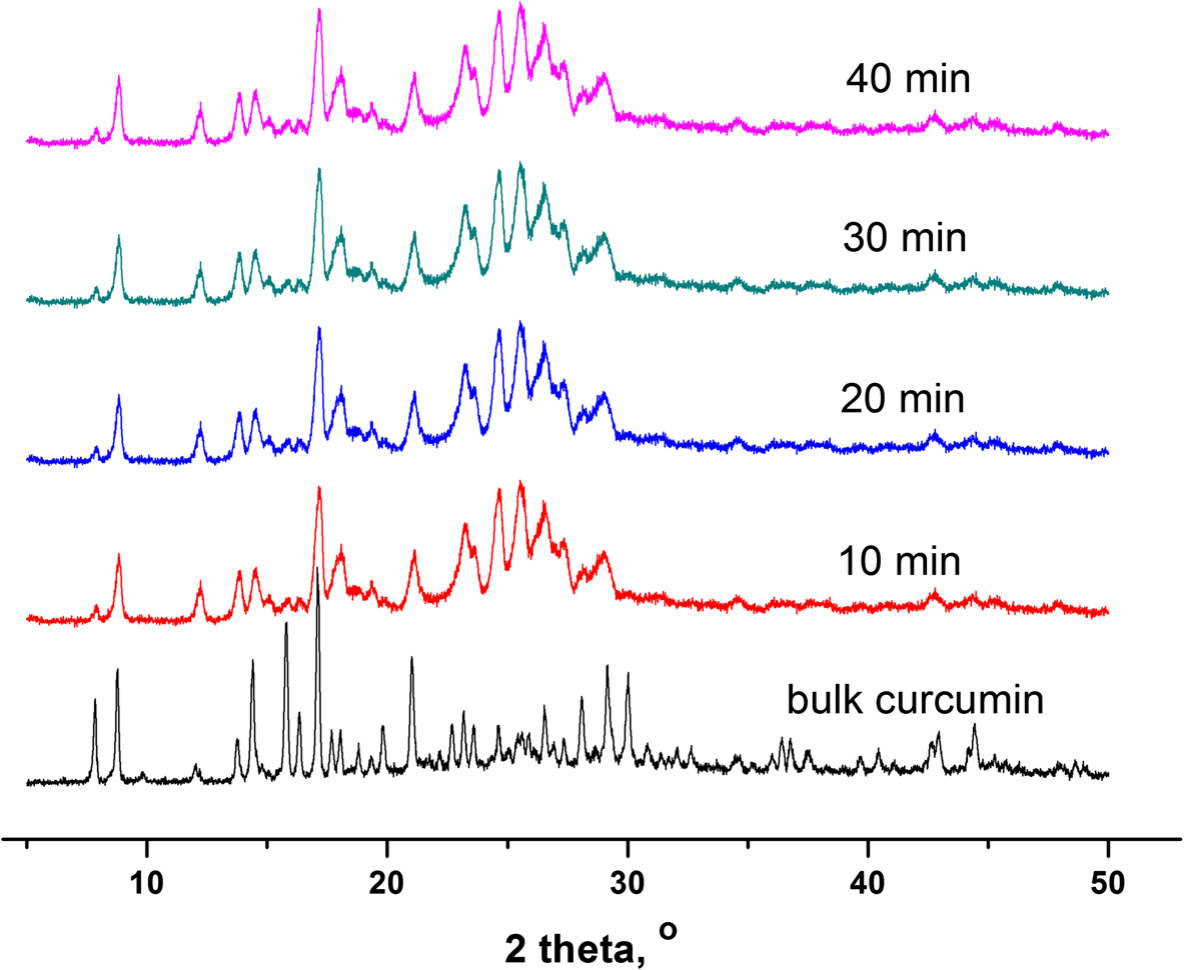
PXRD patterns of bulk curcumin and curcumin-DPIs milled with different times

As seen from the PXRD results in Fig. 4, there were some differences of the polymorphic forms between bulk curcumin and curcumin-DPIs. The bulk curcumin maintained the crystalline state well, while curcumin-DPIs had the lower peak intensities, suggesting the lower crystallinity and the smaller sizes, which was consistent with the SEM results.

#### FTIR Analysis

**Fig. 5:**
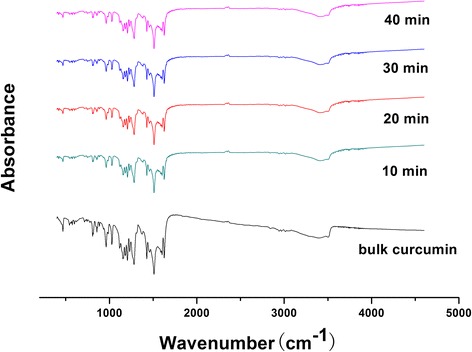
FTIR spectra of bulk curcumin and curcumin-DPIs milled with different times

The chemical compositions of bulk curcumin and curcumin-DPIs were evaluated by FTIR spectra, and it could be seen that there were no obvious differences between the two samples in the whole area of curcumin absorption bands. As shown in Fig. 5, the FTIR analysis showed absorption at 1720 cm^−1^ for C=O stretching of the ester group, at 1650 cm^−1^ for C=O of the ketone group, at 1300 cm^−1^ for the ether group, and at 2980 cm^−1^ for aromatic stretching. A sharp band at about 3500 cm^−1^ and the broad peak at 3200–3500 cm^−1^ in the spectrum have been attributed to the –OH group stretching vibration. It could be concluded that milling and spray drying did not change the chemical compositions of curcumin.

#### Dissolution Test

**Fig. 6:**
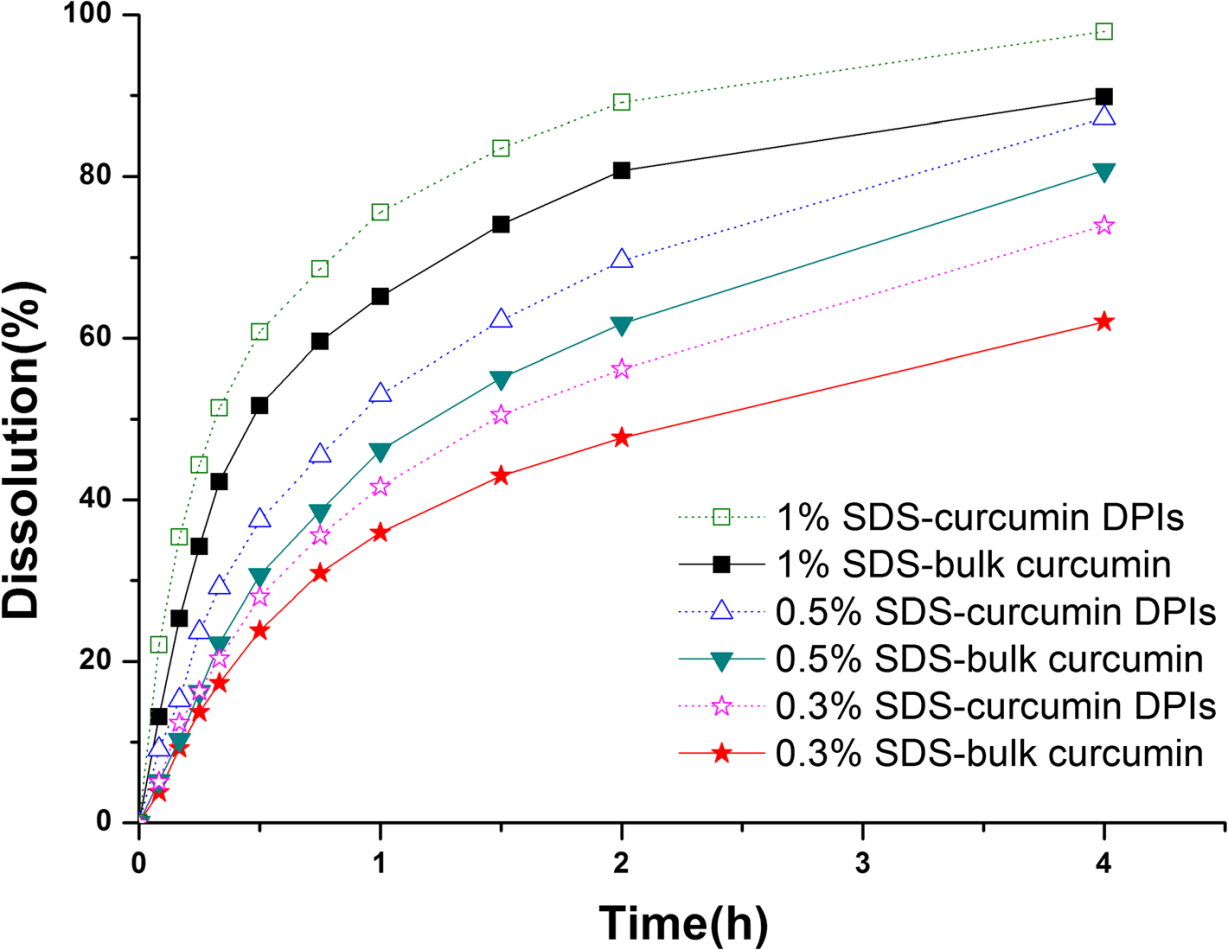
Dissolution profiles of curcumin-DPIs and bulk curcumin in different media

Figure 6 showed the dissolution behavior of bulk curcumin and curcumin-DPIs. Curcumin-DPIs showed a higher dissolution of about 98 % at 4 h with 1 % SDS, 87 % with 0.5 % SDS, and 74 % with 0.3 % SDS, while the dissolution from bulk curcumin was about 90 % with 1 % SDS, 81 % with 0.5 % SDS, and 62 % with 0.3 % SDS. The dramatic increase of dissolution could be attributed to the following reason. According to the Noyes-Whitney equation, the curcumin-DPIs had a smaller size and a larger surface area with a higher apparent saturation solubility, which pronouncedly enhanced the dissolution rate. In addition, the dissolution rate could be improved significantly with the SDS concentration increasing.

#### SEM Analysis

**Fig. 7:**
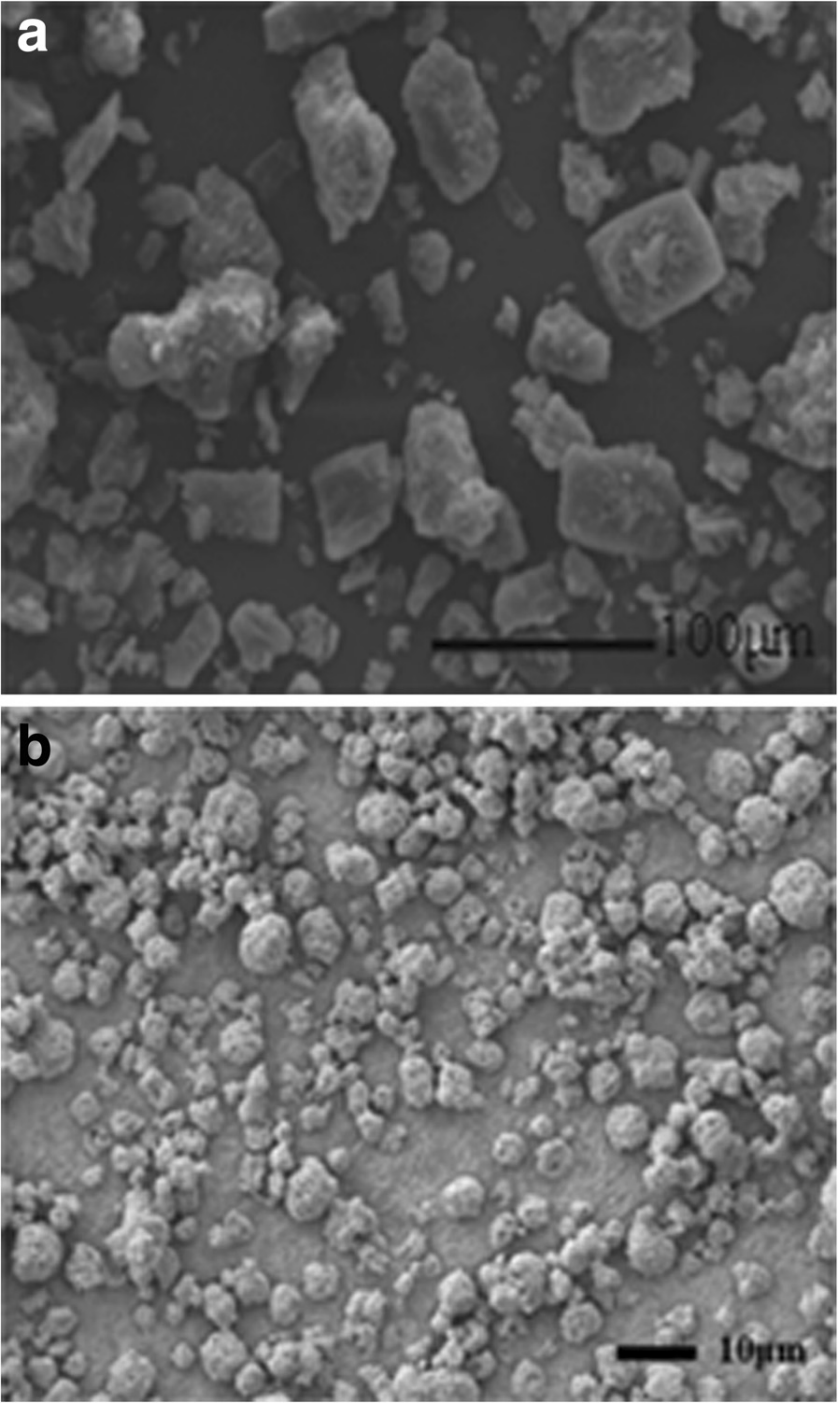
**a** SEM images of bulk curcumin. **b** SEM images of curcumin-DPIs

The SEM results (Fig. 7) presented that the bulk curcumin was in a tabular shape and had a broad particle size distribution. In contrast, the curcumin-DPIs showed spherical, uniform small particle sizes. It was found that some fine crystals adhered with each other during spraying by electrostatic and van der Waals forces, and the particle size became larger. During spray drying, the nanocrystals were sprayed in the form of a lot of small droplets and each droplet consisted of many fine particles. These particles aggregated together after water evaporated instantly, and the aggregates were hard to break into individual particles.

#### Drug Loading and Stability Analysis

The drug loading of curcumin-DPIs was measured. There were no obvious changes of curcumin-DPIs after storage for 60 days. No significant differences about particle size and flowability were found, and the curcumin content remained constant and no degradation peaks were found in HPLC chromatography. Therefore, the curcumin-DPIs showed good physical and chemical stability.

#### Pharmacokinetic and Biodistribution Study

**Fig. 8:**
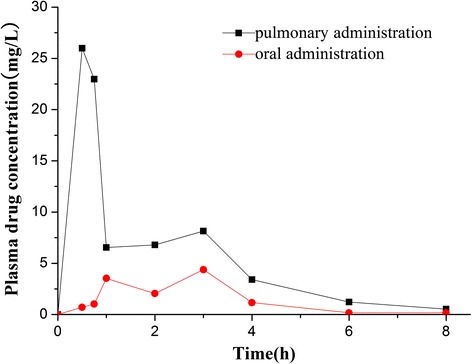
Mean plasma concentration profiles of curcumin-DPIs in rabbits after pulmonary and oral administration

The concentration-time profiles after pulmonary and oral administration are shown in Fig. 8, and pharmacokinetic parameters are summarized in Table 2.

**Table 2:** Mean pharmacokinetic parameters of rabbits after pulmonary and oral administration

Parameters	Pulmonary administration	Oral administration
*T*_1/2_ (h)	1.46	2.15
*C*_max_ (mg/L)	27.52	3.64
AUC (mg L^−1^ h^−1^)	37.24	11.75
MRT (h)	2.65	3.05

The curcumin concentration decrease from the plasma appeared for the pulmonary administration to be in two distinct phases with an initial rapid elimination occurring in the first hour followed by a slower elimination from 1 to 8 h. The first phase probably corresponded to the rapid absorption and distribution of the drug into the systemic compartment until equilibrium was reached between the systemic circulation, the lung, and the different tissues, after which the elimination phase took place. Significant differences were found between the two administration routes. The plasma curcumin concentration was much higher for pulmonary administration than for oral administration.

The average peak concentration (*C*_max_) of the inhalation group (27.52 mg/L) was higher than that of the oral group (3.64 mg/L), and *T*_max_ of the inhalation group was advanced compared with that of the oral group (0.5 versus 3 h), which indicated a greater and faster absorption after pulmonary administration. The shorter *T*_max_ observed for the pulmonary group may also be explained by the higher solubility of curcumin-DPIs. The higher solubility was probably correlated with faster *in vivo* dissolution velocity, as described by the Noyes-Whitney equation, accelerating absorption onset from the lung. AUC_0*–∞*_(area under the curve) of the pulmonary group was 37.24 mg L^−1^ h^−1^, which was about 3.2-fold higher than that of the oral administration group (11.75 mg L^−1^ h^−1^). MRT (mean retention time) of the oral group was 3.05 h, while MRT of the pulmonary group was 2.65 h. Results showed that the curcumin-DPIs could increase the curcumin absorption rate and amount. The bioavailability of curcumin was significantly enhanced by the inhalation delivery.

**Fig. 9:**
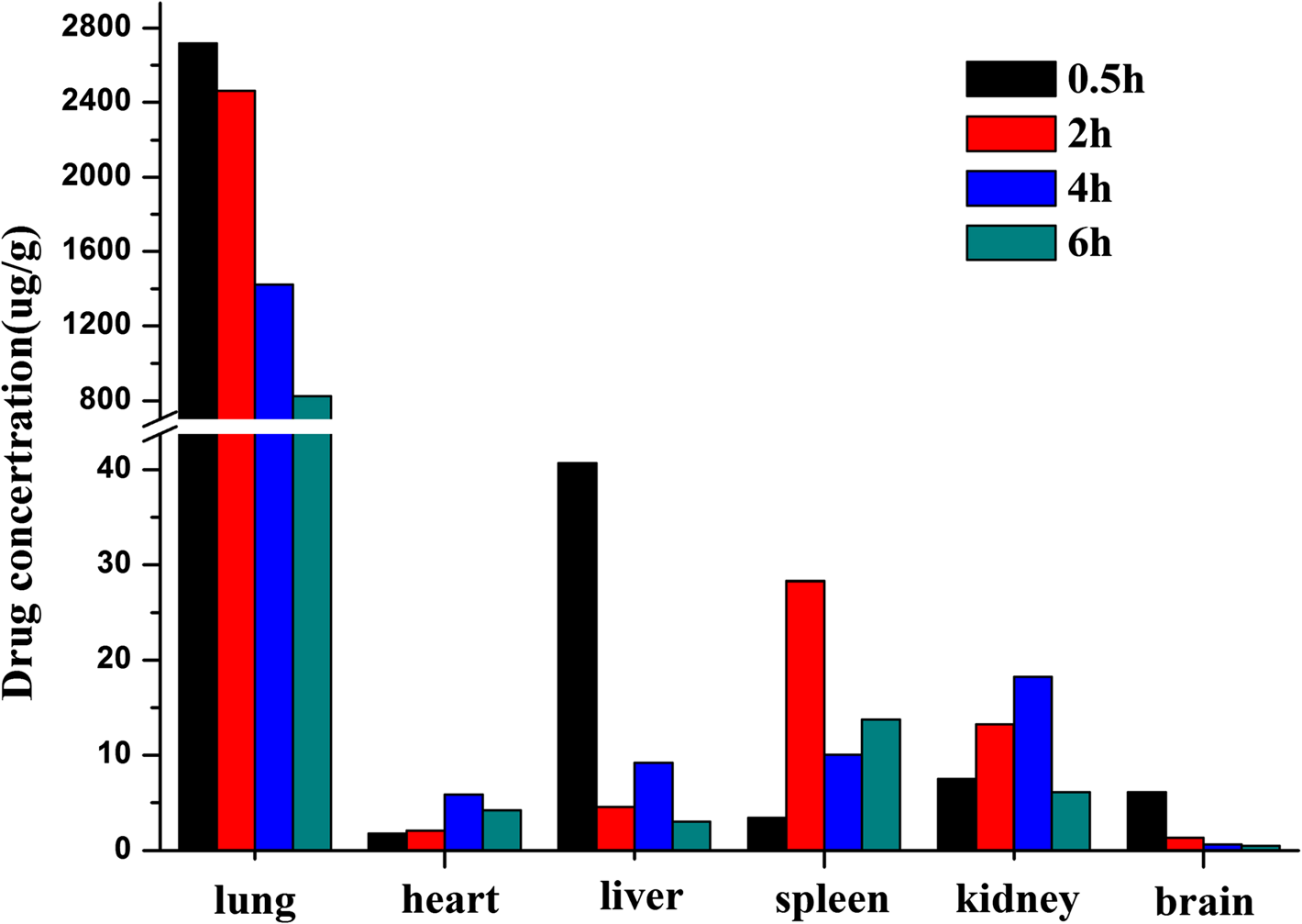
Concentrations of curcumin in tissues following pulmonary administration of curcumin-DPIs

Figure 9 showed the tissue curcumin concentrations of curcumin-DPIs. After pulmonary administration, the distribution of curcumin in the lung was dramatically increased at the predetermined time points compared with other tissues. Six hours after pulmonary administration, curcumin concentration in the lung was still high (824.27 μg/g), while a relatively low distribution in the liver (3.06 μg/g), kidney (6.10 μg/g), heart (4.23 μg/g), spleen (13.77 μg/g), and brain (0.53 μg/g) was found at the same time. The biodistribution study in rats indicated that curcumin-DPIs would mostly be deposited in the lung, which could enhance the lung curcumin concentration and decrease curcumin concentration in other tissues, thus reducing the systemic toxicity and improving the therapeutic effects.

## Conclusions

In this study, curcumin-DPIs were successfully prepared using wet milling technique followed by spray drying. DSC, PXRD, and FTIR tests showed that the crystalline state of curcumin was not changed during the particle size reduction. The aerodynamic performance showed that the milling time of 30 min was most suitable for inhalable administration. The products could be stored for 60 days, and no degradation was found. The pharmacokinetic study demonstrated that curcumin-DPIs could enhance bioavailability of curcumin with a higher AUC and *C*_max_ than those of oral administration. The *in vivo* tissue distribution showed that most of the curcumin-DPIs were deposited in the lung, thus reducing the concentrations in other tissues. Results showed that DPI was a potential drug delivery system for the effective treatment of the lung diseases with improved lung concentration and reduced systematic toxicity.

## Additional file

Additional file 1:
**An additional file showing the particle size with different milling times in more detail.** (XLSX 10 kb)

## References

[1] Gupta SC, Kismali G, Aggarwal BB (2013). Curcumin, a component of turmeric: from farm to pharmacy. Biofactors..

[2] Kumaravel M, Sankar P, Latha P, Benson CS, Rukkumani R (2013). Antiproliferative effects of an analog of curcumin in Hep-2 cells: a comparative study with curcumin. Nat Prod Commun..

[3] Avasarala S, Zhang F, Liu G, Wang R, London SD, London L, et al. Curcumin modulates the inflammatory response and inhibits subsequent fibrosis in a mouse model of viral-induced acute respiratory distress syndrome. Plos One. 2013;8:e57285. 10.1371/journal.pone.0057285 PMC3577717 23437361

[4] Peng F, Tao Q, Wu X, Dou H, Spencer S, Mang C (2012). Cytotoxic, cytoprotective and antioxidant effects of isolated phenolic compounds from fresh ginger. Fitoterapia..

[5] Lim KJ, Bisht S, Bar EE, Maitra A, Eberhart CG (2011). A polymeric nanoparticle formulation of curcumin inhibits growth, clonogenicity and stem-like fraction in malignant brain tumors. Cancer Biol Ther..

[6] Anand P, Kunnumakkara AB, Newman RA, Aggarwal BB (2007). Bioavailability of curcumin: problems and promises. Mol Pharm..

[7] Wei X-L, Han Y-R, Quan L-H, Liu C-Y, Liao Y-H. Oily nanosuspension for long-acting intramuscular delivery of curcumin didecanoate prodrug: preparation, characterization and *in vivo* evaluation. Eur J Pharm Sci. 2013;49:286–93. 10.1016/j.ejps.2013.03.010 23542494

[8] Jain RA, Brito L, Straub JA, Tessier T, Bernstein H (2008). Effect of powder processing on performance of fenofibrate formulations. Eur J Pharm Biopharm..

[9] Liu P, Rong X, Laru J, Veen B van, Kiesvaara J, Hirvonen J, et al. Nanosuspensions of poorly soluble drugs: preparation and development by wet milling. Int J Pharm. 2011;411:215–22. 10.1016/j.ijpharm.2011.03.050 21458552

[10] Courrier H, Butz N, Vandamme TF (2002). Pulmonary drug delivery systems: recent developments and prospects. Crit Rev Ther Drug Carrier Syst..

[11] Xu L-M, Hu T-T, Pu Y, Le Y, Chen J-F, Wang J-X, et al. Preparation of high-performance ultrafine budesonide particles for pulmonary drug delivery. Chem Eng J. 2014;252:281–7.

[12] Rohani SSR, Abnous K, Tafaghodi M (2014). Preparation and characterization of spray-dried powders intended for pulmonary delivery of insulin with regard to the selection of excipients. Int J Pharm..

[13] Klingler C, Müller BW, Steckel H (2009). Insulin-micro-and nanoparticles for pulmonary delivery. Int J Pharm..

